# Variations in geographical accessibility: a cross-sectional nationwide study of travel times in Swedish primary care

**DOI:** 10.1136/bmjopen-2026-120220

**Published:** 2026-09-11

**Authors:** David Isaksson, Sofie Vengberg, Mio Fredriksson

**Affiliations:** 1 Health Services Research, Department of Public Health and Caring Sciences, Uppsala University, Uppsala, Sweden

**Keywords:** Health Services Accessibility, Primary Care, Health Equity, Geographical mapping, General Practice

## Abstract

**Abstract:**

**Objectives:**

To examine geographical accessibility to primary care in Sweden by estimating driving distances and travel times to primary care centres, assessing the availability of alternative providers within predefined travel-time thresholds and describing variation by municipality type.

**Design:**

Descriptive cross-sectional nationwide study.

**Setting:**

Primary care in Sweden, including all primary care centres operating nationally during 2019–2020.

**Participants:**

Individual-level residential grid coordinates for all residents of Sweden as of 31 December 2019 were obtained. After exclusion of observations with missing coordinate data, the final analytical sample comprised 9 946 509 individuals. A total of 1167 primary care centres were included after merging duplicate administrative units at the same physical location.

**Outcome measures:**

Outcomes were driving distance and driving travel time by car from place of residence to the nearest primary care centre. Furthermore, driving distance and travel time to the second- to fifth-nearest centres, and the number of centres reachable within 5, 10, 20 and 30 min by car were used as outcomes.

**Results:**

Nationally, the mean travel distance and time to the nearest primary care centre were 3.84 km (SD=5.8) and 5.6 min (SD 6.3), respectively (median distance 1.63 km, IQR 0.90–3.67; median time 3.3 min, IQR 2.0–6.2). Overall, 15.8% of residents could not reach a primary care centre within 10 min by car while 0.9% had travel times exceeding 30 min. Mean travel time to the nearest centre was 15.1 min (SD 18.9) in very rural municipalities compared with 3.0 min (SD 2.5) in large cities.

**Conclusions:**

Geographical accessibility to Swedish primary care is high on average but substantial urban–rural disparities remain. Residents of sparsely populated areas face markedly longer travel times and more limited practical provider choice. Future studies should complement geographical measures with indicators of staffing, waiting times and multimodal transport to better assess medical deserts and effective access.

**STRENGTHS AND LIMITATIONS OF THIS STUDY**

- This study uses nationwide, individual-level data covering the entire Swedish population, enabling a comprehensive assessment of geographical accessibility without sampling bias.
- High-resolution residential grid data allowed detailed spatial analysis while preserving individual anonymity.
- Travel distances and times were calculated using a validated routing engine based on real road network data, improving accuracy compared with straight-line measures.
- Travel times were estimated for car transport only, potentially overestimating accessibility for individuals without access to private vehicles or in areas with limited transport infrastructure.
- The analysis includes only primary care centres and does not account for satellite clinics, outreach services or home healthcare, which may lead to underestimation of accessibility in some areas.

## Introduction

 From a global perspective, there has been a growing emphasis on primary care in recent decades, and the WHO advocates for a reorientation of healthcare systems to primary care.^1^ This increased focus can be attributed to several factors. First, a comprehensive primary care sector is considered an efficient way of promoting equitable health outcomes in the population. Second, it is widely recognised that primary care offers a more cost-efficient way of delivering healthcare services.^2^ Primary care is the first contact, the main entry point for people into the healthcare system, and constitutes an interface between people and the healthcare system.^3^ Consequently, primary care assumes a pivotal role in safeguarding the overall health of the population, not the least because of its focus on health promotion, disease prevention and addressing health determinants.

To fulfil this role, it is essential that the entire population has adequate access to primary care. Access comprises several dimensions, in terms of both accessibility of healthcare services and patients’ abilities to reach and use them.^4 5^ Dimensions of accessibility include aspects such as timeliness, geographical accessibility and affordability. In this article, the focus is on *geographical accessibility* and the related concept of *geographical equity*.^6^ Geographical equity encompasses both demand-side determinants (eg, transportation resources, mobility, social support) and supply-side determinants (eg, the spatial distribution of providers, opening hours and appointment mechanisms).^4^ Our analysis concentrates on the supply-side perspective, ie *geographical accessibility,* examining the spatial distribution of primary care centres in relation to the Swedish population. Specifically, we measure *travel distances* and *travel times*, two key indicators of geographical accessibility.

This focus also relates to the emerging concept of *medical deserts*—geographical areas where populations face significant challenges in accessing primary care. Medical deserts are typically defined by a combination of factors, including long distances to healthcare facilities, extended travel times and insufficient provider capacity or staffing.^7–9^ However, definitions and thresholds vary across countries depending on geographical conditions, urban–rural classifications and the structure of the healthcare system.^8^ In the present study, we focus on the geographical dimension of medical deserts by analysing travel distances and travel times to primary care centres across Sweden.

All countries face some challenges related to geographical imbalances in, for example, human resources, which affect geographical equity^9 10^ as well as the distribution of primary care resources.^11^ Geographical variation is also noticed in terms of both utilisation and health outcomes, often linked to an urban–rural dimension where spatial barriers indicate lower utilisation.^12 13^ For example, studies have shown that rural residents are more likely than their urban counterparts to face multiple system-level barriers to accessing medical care^14^ and that they use primary care to a lesser extent.^15^ In specialised care, there is a clear association between health outcomes and patient travel time.^16^ Several studies have found that regional variations in healthcare utilisation are to a greater extent explained by place factors than by needs or socioeconomic factors, especially within primary care.^17 18^ This represents a type of geographical inequity, stemming not from area effects on health or healthcare utilisation but area effects on healthcare production and service delivery.^6^ Yet, there are mitigating factors, most notably the use of digital solutions.^19 20^ Reimbursement systems can also be used to steer more providers to establish in more non-urban areas, for example by allocating additional reimbursement to providers located in rural areas.^6^

Research investigating variations in travel distances and travel times to primary care providers across entire countries is unusual. Comparing results between countries is challenging due to differences in factors such as geographical conditions, public expectations, health characteristics and the organisation and responsibilities of primary care. For the same reason, it is equally challenging to compare the extent of medical deserts across countries. Nonetheless, research from Germany suggests that in about 90% of cases, primary care physicians are reached within 30 min,^21^ while 13% of the population resides in areas with poor accessibility.^22^ In the UK, Bauer *et al*^23^ calculate that the average distance to the nearest GP is 2.2 min by car but simultaneously indicate that 25.8% of the population lives in areas with a low GP accessibility. Recent research from Finland, which is potentially most comparable to Swedish conditions, suggests that 13% of the population reside in medical deserts^9^ but that about 76% of the population reaches a health centre by car in 10 min and 96% in 20 min.^12^

Inadequate accessibility has been identified as one of the most prominent weaknesses in the Swedish healthcare system.^24^ While this issue is more acute in hospital care—characterised by long waiting times for visits and treatments—inadequate accessibility in primary care has also been a long-standing concern. In response, Sweden introduced waiting-time guarantees in 2010, 2015 and 2022. Another response was a national market-oriented reform implemented in 2010, which mandated all 21 regions to implement a patient choice system in primary care that introduced free establishment for private providers. Key objectives of the reform were to improve access and provide patients with enhanced choice of providers.^25^ Studies suggest that overall access has improved, reflected in an increased number of providers and, in some regions, a rise of visits to general practitioners.^26^

However, the geographical distribution of these improvements in accessibility remains less clear.^27^ What can be observed is that there is a difference in localisation patterns in rural and urban areas. Thus, individuals’ travel distances and travel times to primary care will likely vary greatly depending on geographical conditions, but to what extent is unknown. Today, only national averages calculated by The Swedish Agency for Economic and Regional Growth in 2014 and 2018 are known. In 2014, 97% of the Swedish population had a shorter driving distance than 20 min to the nearest primary care centre.^28^ In 2018, as many as 91% had a shorter driving distance than 10 min and ~1% had a longer driving distance than 20 min.^29^ These figures indicate good geographical accessibility in Swedish primary care on average, but say nothing about geographical equity. Sweden is a country that encompasses both highly urban and highly rural areas. It is among the least densely populated countries in Europe (25.9 persons per square kilometre), but regional differences are large (2–378 persons per square kilometre). The most sparsely populated region, with only two persons per square kilometre, covers almost 25% of the area in Sweden. As such, considerable variation in geographical accessibility to primary care services relating to the urban–rural dimension is to be expected. However, the extent of this geographical variation remains largely unexplored. A nationwide description of geographical accessibility is needed to provide an empirical baseline for healthcare planning, the evaluation of future structural reforms and future studies of geographical inequalities in access to primary care. This is particularly important because previous research has shown that distance is one of the most important factors influencing patients’ choice of primary healthcare provider.^30^

Hence, the aim of this article is to examine variations in the geographical accessibility of primary care in Sweden. We set out to answer the following research questions: What are the travel times and travel distances for residents in Sweden to their nearest primary care centre? How many primary care centres are located within predefined travel time thresholds for residents in Sweden? And how does travel time vary between and within municipalities with different degrees of urbanity?

## Methods

### Setting

The Swedish healthcare system is organised into 21 regions, each with a high degree of autonomy, including taxation authority and its own governing body. The national government primarily exerts influence over the sector through legislation and other regulatory measures. Since 2010, all regions have been mandated by the national government to implement a patient choice system within primary care. This system enables all patients to choose and register with their preferred primary care centre, whether privately or publicly owned, which are reimbursed based on patients’ choices. While the specific reimbursement systems are determined by and vary across regions, the majority rely primarily on capitation-based reimbursements.^31^ Regions cannot decide the location of private primary care centres, but can—and do—design reimbursement systems to compensate both public and private providers located in less urban areas, potentially incentivising establishments in such areas.^27^ Approximately half of all primary care centres are publicly owned by the regions, while the other half is privately owned, largely by for-profit entities.^27^ In comparison to many other countries, Swedish primary care providers tend to be relatively large, both in terms of the number of registered patients and staff size, and are typically staffed by multi-professional teams, comprising physicians, registered nurses, auxiliary nurses, child healthcare specialists and physiotherapists.^32^

### Design and data

This study employs a descriptive cross-sectional design, using individual-level data for all residents of Sweden, with data from 2019 to 2020, along with data on all primary care providers operating in the country during that period.

#### Individual data

Statistics Sweden, the national government agency responsible for official statistics, provided individual-level data, capturing the coordinates of each individual’s place of residence as of 31 December 2019. To safeguard individual anonymity, exact coordinates were not collected. Instead, locations were aggregated into 250-metre grid squares in urban areas and 1000-metre squares for rural areas, placing coordinates in the centre of the square. The dataset was further categorised by municipality type, based on classifications from The Swedish Agency for Economic and Regional Growth.^28^ Observations that had missing coordinate data were excluded. This resulted in a sample of 9 947 224 individual observations, which was used in the primary analysis when calculating driving distances and travel times.

#### Primary care centre data

Information on primary care centre addresses was collected from the official websites of all providers and coordinates were calculated based on these addresses (n=1170). During collection, we noticed that some providers, although administratively listed as separate units, were in fact the same providers, operating from the same physical location and with the same manager. These providers were merged in the analysis. The final sample therefore included 1167 providers.

### Analysis

#### Calculating travel distances and travel times

In this study, travel distances and travel times are operationalised as driving distances and driving travel times by car. To calculate these measures for all individuals to all primary care centres, we used the OSRM package V.4.2.0 in R,^33^ which uses the Open Source Routing Machine, an open-source routing engine that computes driving distances and travel times based on road network data from OpenStreetMap. Travel times were estimated using the default routing profile in OSRM based on the road network and road-specific speed assumptions in OpenStreetMap. Estimated travel times therefore represent expected travel under typical driving conditions and do not account for congestion, weather conditions or temporary traffic disruptions. As previously noted, individual coordinates were derived from 250-metre and 1000-metre grid squares, with coordinates representing the centroid of each grid square. In some cases, this resulted in individuals being located in bodies of water, placing them outside municipal boundaries. To address this issue, these individuals were reassigned to the coordinates of the nearest road in order to enable calculation of driving routes.

During the calculation of driving travel times, we identified extreme outliers, with some individuals having estimated travel times of up to 16 hours by car to their nearest primary care centre. Closer inspection showed that these cases involved individuals residing on islands, where the routing engine had difficulties generating accurate car-based travel time estimates. As it is reasonable to assume that individuals living on islands are likely to use boats rather than cars for travel, these observations were excluded. In total, 715 individuals were removed from the analysis, resulting in a final sample of 9 946 509 individuals.

#### Statistical analysis

Statistical analyses comprised descriptive summaries of travel distances and driving travel times to the five nearest primary care centres. For each individual, we calculated measures of central tendency and dispersion, including mean, median and selected percentiles. In addition, we computed the number of primary care centres reachable within 5-, 10-, 20- and 30-min driving time thresholds. Results were stratified by municipality type according to the classification developed by the Swedish Agency for Economic and Regional Growth,^28^ enabling comparisons across different settlement structures. Descriptive results were presented using tables, figures and thematic maps. Graphical visualisations were produced using ggplot2 V.3.5.2^34^ and maps were created using the tmap package.^35^ All statistical analyses and visualisations were conducted in R V.4.5.2.^36^ As the study was based on complete population data rather than a sample, inferential statistical measures such as CIs and hypothesis tests were not applied.

### Reporting

We used the Strengthening the Reporting of Observational Studies in Epidemiology (STROBE) reporting checklist^37^ to guide the reporting in this manuscript (online supplemental A).

### Patient and public involvement

Patients or the public were not involved in the design, conduct or reporting of this research. However, the results will be disseminated to relevant patient organisations to support awareness and discussion of geographical accessibility to primary care.

## Results

The results are structured to first describe overall accessibility patterns, followed by distributional and time-threshold analyses, and finally geographic variation by municipality and municipality type.

### Overall distances and travel times

There is noticeable variation among individuals in terms of the distance to their nearest primary care provider. For the nearest primary care centre, the median travel distance was 1.63 km (IQR 0.90–3.67), with a mean of 3.84 km (SD 5.8). The maximum observed distance was 182.8 km, while the 99th percentile reached 25.9 km. In contrast, the median distance to the second-nearest primary care centre was 3.29 km (IQR 1.80–11.3), with a mean of 8.21 km (SD 11.3) and a 99th percentile of 53.0 km, see table 1.

**Table 1:** Summary statistics on travel distances and travel times to the five nearest primary care centres for all individuals residing in Sweden

	Mean (SD)	Min	p01	p25	p50	p75	p99	Max
Travel distances (km)								
Nearest	3.84 (5.8)	0.00	0.14	0.90	1.63	3.67	25.9	182.8
Second nearest	8.21 (11.3)	0.03	0.51	1.80	3.29	11.3	53.0	214.7
Third nearest	11.2 (14.9)	0.27	0.87	2.48	4.91	15.7	74.7	251.3
Fourth nearest	13.8 (16.8)	0.62	1.11	3.10	6.77	19.7	85.4	281.0
Fifth nearest	16.4 (19.6)	0.80	1.29	3.69	9.30	23.5	94.2	311.6
Travel time by car (minutes)								
Nearest	5.6 (6.3)	0	0.4	2.0	3.3	6.2	29	230
Second nearest	10.2 (10.8)	0.1	1.3	3.7	6	14	53	232
Third nearest	13.2 (13.6)	0.7	2.0	4.8	8	18	70	250
Fourth nearest	15.8 (15.3)	1.2	2.4	5.7	10	21	78	307
Fifth nearest	18.0 (17.4)	1.6	2.9	6.5	12	25	88	326

Distances increased progressively for the third-, fourth- and fifth-nearest primary care centres, with median distances of 4.91 km (IQR 2.48–15.7), 6.77 km (IQR 3.10–19.7) and 9.30 km (IQR 3.69–23.5), respectively. The corresponding mean distances were 11.2 km (SD 14.9), 13.8 km (SD 16.8) and 16.4 km (SD 19.6), with maximum values reaching up to 311.6 km for the fifth-nearest provider.

Corresponding patterns were observed for travel times. The median travel time to the nearest primary care centre was 3.3 min (IQR 2.0–6.2), with a mean of 5.6 min (SD 6.3) and a maximum of 230 min. For the second-nearest primary care centre, the median travel time was 6 min (IQR 3.7–14 min), with a mean of 10.2 min (SD 10.8) and a 99th percentile of 53 min. Travel times continued to increase for the third-, fourth- and fifth-nearest primary care centres, with median values of 8 min (IQR 4.8–18), 10 min (IQR 5.7–21), and 12 min (IQR 6.5–25), and corresponding mean values of 13.2 min (SD 13.6), 15.8 min (SD 15.3), and 18.0 min (SD 17.4), respectively.

These results illustrate a clear gradient in accessibility, where travel distances and times increase substantially beyond the nearest provider, particularly in the upper percentiles. This suggests considerable geographic variation in accessibility to the second-nearest primary care provider. For example, while the 75th percentile travel time to the nearest primary care centre was just 6 min—which could be considered negligible—there is a considerable jump to 14 min for the second-nearest provider.

**Figure 1:**
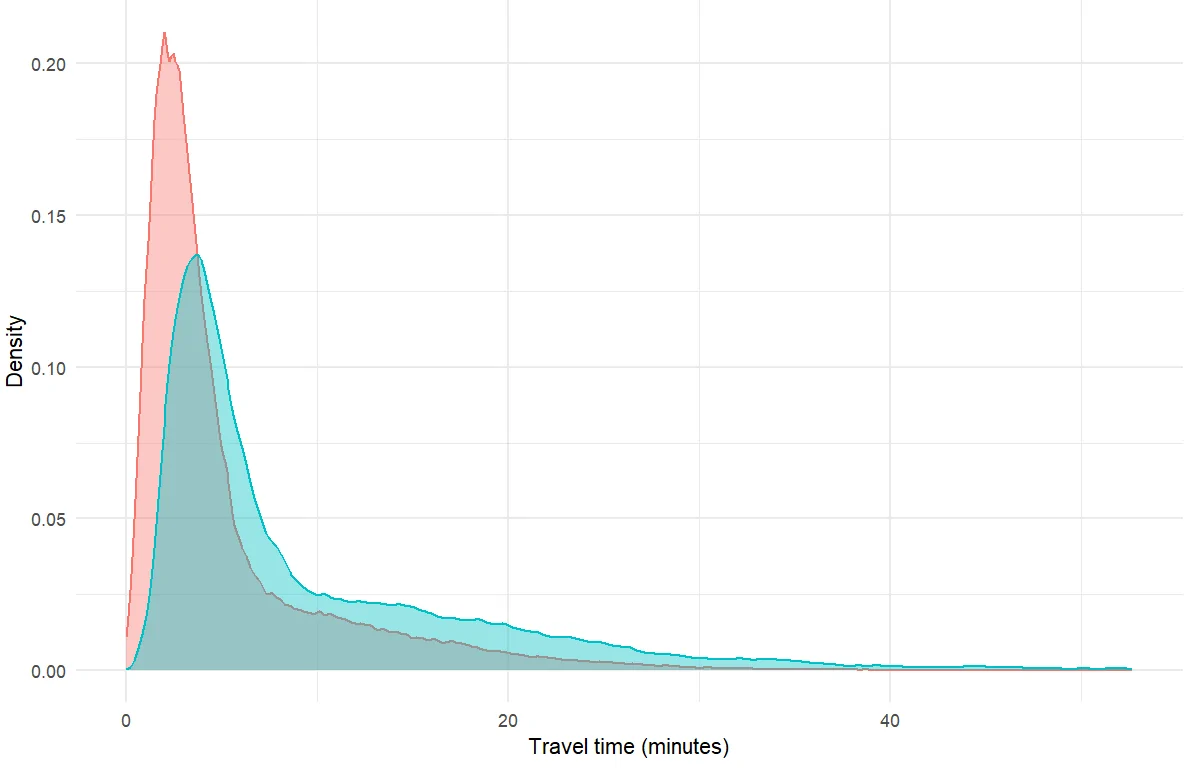
Distribution of travel time to the nearest and second-nearest primary care centres. Only showing the 99% of the population with the shortest travel time. Red area indicates travel time to nearest primary care centre, blue area indicates travel time to second-nearest primary care centre.

To explore this distribution in greater detail, travel times are presented in two distribution plots. Figure 1 illustrates the distribution of travel times to the nearest and second-nearest primary care centre, excluding the 1% of individuals with the longest travel times. As shown in the figure, the large majority of residents in Sweden can reach both their nearest and second-nearest primary care centre by car within a relatively short time frame.

**Figure 2:**
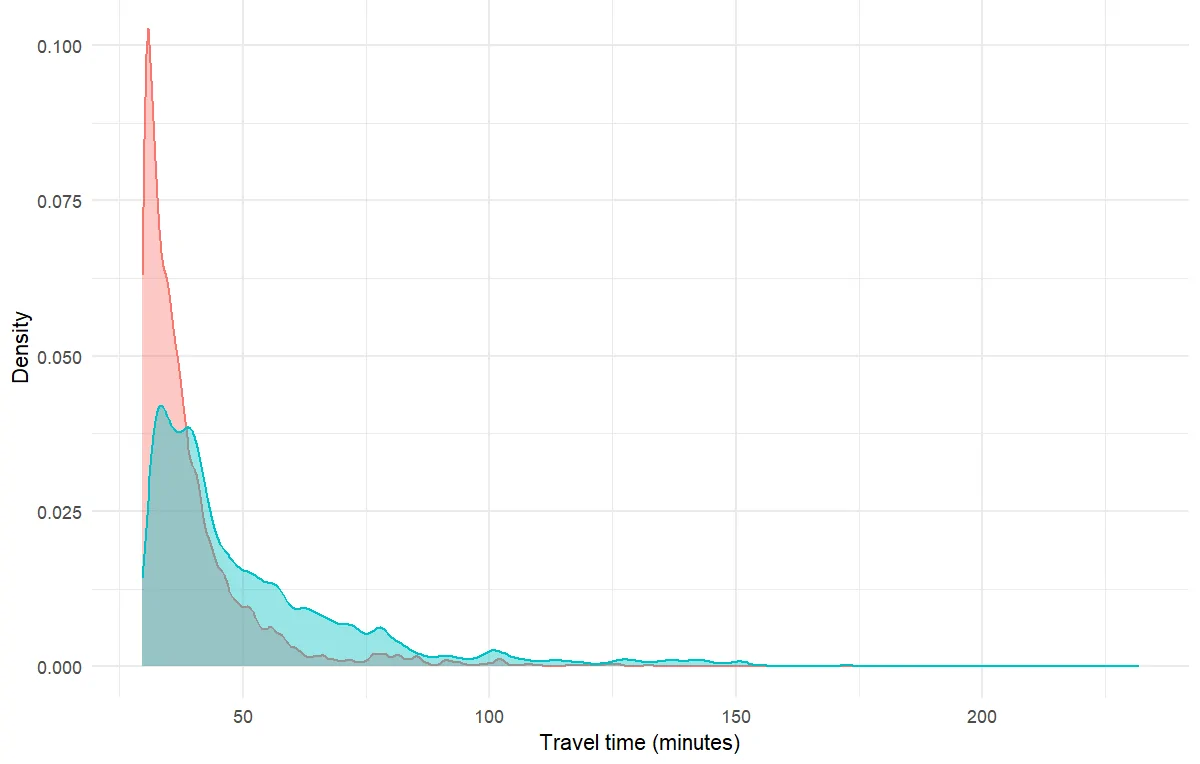
Distribution of travel time to nearest and second-nearest primary care centres. Only showing the 1% of the population with the longest travel times. Red area indicates travel time to nearest primary care centre, blue area indicates travel time to second nearest primary care centre.

Figure 2 shows the variation in travel times among the 1% of the population with the longest travel times to their nearest primary care centre—approximately 100 000 individuals with travel times exceeding 29 min.

### Number of primary care centres within predefined time thresholds

**Figure 3:**
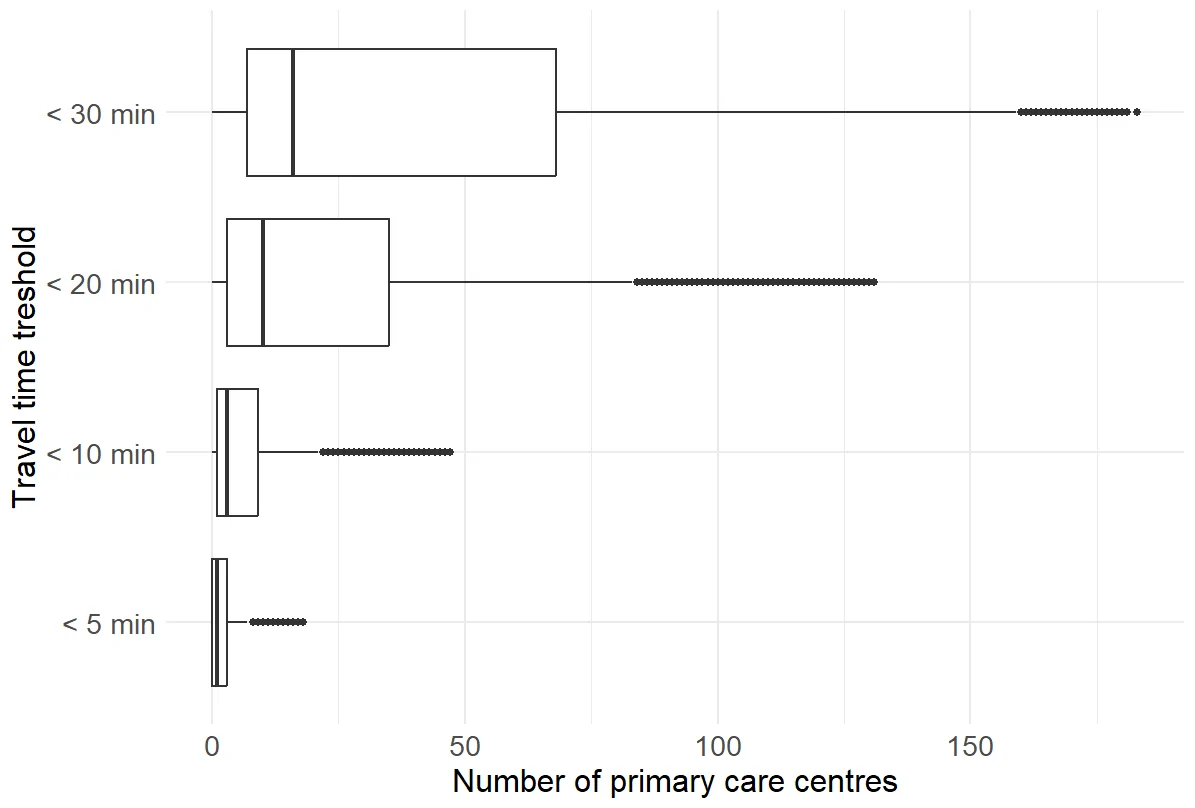
Number of primary care centres reachable within certain travel time thresholds.

To complement the analysis of travel times, we also examined the number of primary care centres accessible within specific time intervals. Figure 3 presents a boxplot illustrating the number of primary care centres an individual can reach within specific travel time thresholds. As illustrated in the figure, a majority of Swedish residents can reach several primary care centres within 20 min by car.

There are, however, some residents that cannot reach a primary care centre within these travel time thresholds. Almost 16% of Swedish residents are not able to reach a primary care centre within 10 min travelling by car whereas 0.9% have a travel time by car exceeding 30 min to their nearest primary care centre, see table 2.

**Table 2:** Residents unable to reach a primary care centre by car within certain time thresholds

Travel time threshold	Residents unable to reach a primary care centre	
	Number	Percentage (%)
<5 min	3 152 371	31.7
<10 min	1 570 819	15.8
<20 min	390 520	3.9
<30 min	92 431	0.9

**Figure 4:**
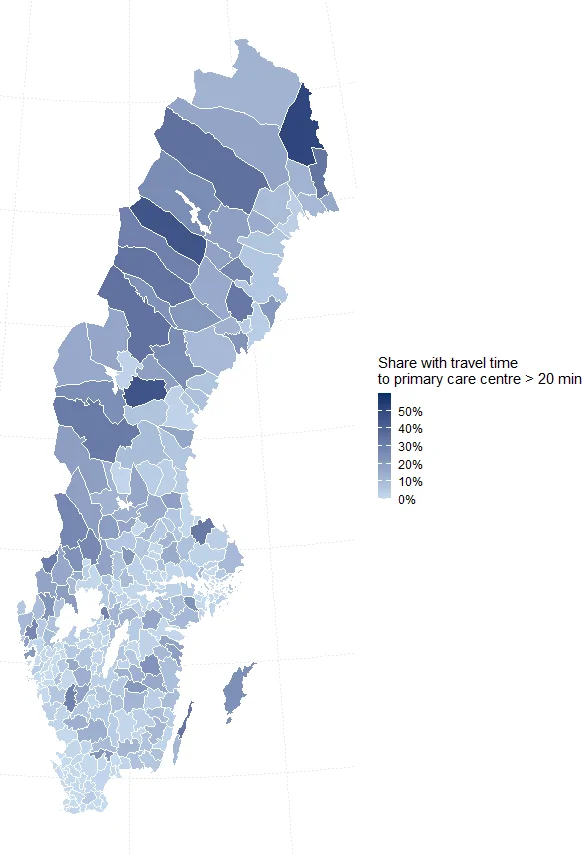
Share of individuals within each municipality with a travel time of 20 min or more to their nearest primary care centre.

The travel times are not evenly distributed across the country. Certain parts of Sweden have a higher proportion of individuals with longer travel times to their nearest primary care centre. To illustrate this, figure 4 shows the share of individuals within each municipality who have a travel time of 20 min or longer to their nearest primary care centre.

### Travel times related to municipality type

**Figure 5:**
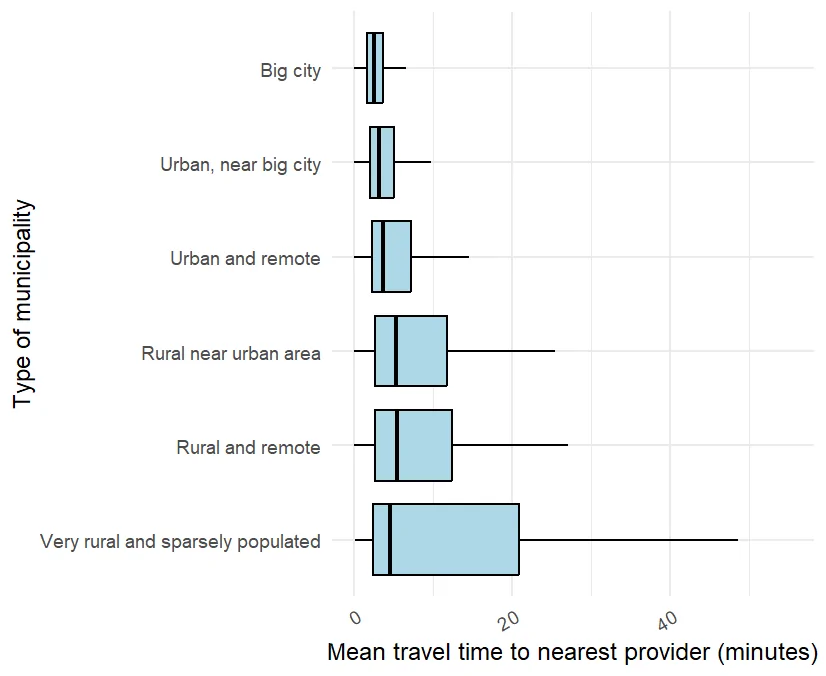
Travel time to nearest primary care centre, grouped by municipality type.

An additional consideration is whether the observed variation in travel times can be attributed to municipality type, in terms of degree of urbanity or rurality. To examine this, we created a histogram depicting the travel times by municipality type, as presented in figure 5. Individuals living in very rural and sparsely populated municipalities had longer travel times to their nearest primary care centre, with a mean of 15.1 min (SD 18.9) and a median of 4.8 min (IQR 2.4–23.6), compared with those in urban and remote municipalities, with a mean of 7.0 min (SD 8.1) and a median of 4.0 min (IQR 2.4–9.0) and residents of big cities with a mean of 3.0 min (SD 2.5) and a median of 2.6 min (IQR 1.7–3.8). Furthermore, figure 5 illustrates a clear trend: the more urban the municipality in which an individual resides, the more likely residents are to have shorter travel times to the nearest primary care centre. Notably, the median values do not differ substantially across municipality types; rather, the differences are reflected in the proportion of individuals with longer travel times within each municipality type.

## Discussion

Notably, a substantial proportion of the Swedish population resides relatively close to their nearest primary care centre, with a national average of 3.84 km and 5.6 min in travel distance and time, respectively. However, considerable variation in geographical accessibility is observed across the country. Much of this variation can be attributed to the degree of urbanisation in the area where an individual resides. For example, individuals living in very rural and sparsely populated municipalities travel an average of 15 min by car to reach their nearest primary care centre, compared with 7 min in urban and remote municipalities and 3 min in big cities. Importantly, differences also exist within groups of municipalities with the same degree of urbanisation—with some rural municipalities exhibiting relatively short average travel times.

Among individuals in the top 1% in terms of longest distance to their nearest primary care centre, there is substantial variation in travel times. Some face journeys of up to 4 hours by car, while others in this extreme group experience travel times as short as 29 min, despite being in the 99th percentile of travel distance. This highlights the skewed distribution of accessibility among those residing farthest from care. For the vast majority of the population, however, both travel distances and travel times to the nearest and second-nearest primary care centre are relatively short, suggesting generally good geographical accessibility.

The possibility of choosing between different primary care centres, defined as having multiple providers within a reasonable distance, follows a similar pattern, with even more pronounced differences between individuals with short and long travel times. In many cases, residents in remote areas may lack a practical choice of provider. Variation in distance to the second-nearest primary care centre is greater than for the nearest provider, which has implications for patients seeking to switch providers. In such instances, the opportunity to do so is limited in certain areas, thereby restricting patients’ ability to change primary care centre if dissatisfied with treatment or care quality.

This raises an important question: what constitutes a reasonable travel time to primary care? In rural or sparsely populated regions, longer travel times may be unavoidable, and to a certain degree also accepted by residents.^38^ While there is no universally accepted threshold, previous research and policy guidelines have proposed various benchmarks. Travel times of a maximum of 30 min are often cited as a standard for acceptable proximity to primary care services.^39 40^ Earlier estimates by The Swedish Agency for Economic and Regional Growth,^28^ along with our results, indicate that very few residents in Sweden cannot access primary care within 30 min by car. Beyond 30 min, growing evidence suggests that longer travel times can reduce the likelihood of patients seeking timely care, particularly for preventive or non-urgent services,^15 41 42^ which may adversely affect health outcomes.^16^ Other studies suggest lower thresholds—ranging from 10 to 20 min—as more appropriate, particularly in urban or densely populated areas where expectations of accessibility may be higher and infrastructure supports shorter travel times.^43^

In this study, most individuals had access to at least one primary care centre within 10 min travel time, and many had multiple providers within a 15–20 min range, indicating a strong baseline level of accessibility. However, the wide variation among the 1% with the longest distance to the nearest primary care centre underscores the limitations of relying solely on averages when assessing geographic equity in accessibility. For planning and policy purposes, targeted efforts may be needed to support individuals in outlier areas where travel times exceed accepted thresholds. More broadly, the results may support regional planning by identifying municipalities with particularly long travel times and by providing a national benchmark for evaluating future changes in primary care infrastructure.

Findings from other Western European countries point in a similar direction.^21–23^ Direct international comparisons should nevertheless be made with caution, as organisational models differ substantially. Sweden relies on large, multi-professional *primary care centres* rather than single-physician GP practices, which enhances comprehensiveness but limits the number of sites. This structure makes it more difficult to achieve full geographical coverage, meaning that some Swedish municipalities likely meet criteria associated with *medical deserts*—long travel times, long travel distances and limited staffing—despite overall good national accessibility.

From an international perspective, the Nordic countries offer the most relevant comparison to Sweden due to their similar geography, population density and welfare systems. Recent studies from Finland, a country that similar to Sweden uses multi-professional primary healthcare centres, show that 76% of residents can reach a health centre within 10 min by car and 96% within 20 min, while approximately 13% live in areas classified as *medical deserts*.^9 12^ In Sweden, 84% of the population can reach a primary care centre within 10 min and fewer than 1% face travel times exceeding 30 min, indicating slightly better overall geographical accessibility. However, both countries exhibit pronounced urban–rural disparities, suggesting shared challenges in ensuring equitable access across sparsely populated areas.

It is important to recognise that geographical accessibility represents only one dimension of healthcare accessibility. Although a necessary condition, it is not sufficient to ensure comprehensive accessibility.^4^ Although our results indicate that most individuals in Sweden have good geographical accessibility, this does not automatically translate into overall accessibility in primary care. Even when geographical distances are short, long waiting times—often driven by staffing challenges—and limitations in public transportation can negatively affect geographical accessibility. To be able to properly define medical deserts in Sweden, we need to add data on either staffing, waiting times or number of visits. If primary care centres are close but understaffed with long waiting times, accessibility is poor, which may be the case also in urban (often deprived) areas.^44^

For those living far from any primary care centre, alternative solutions for healthcare delivery may be necessary. Digital primary care, for instance, has the potential to benefit this group and help alleviate the challenges associated with inadequate geographical accessibility.

### Limitations

One limitation of this study is that we have only calculated distances to primary care centres. In some instances, primary care providers may operate smaller satellite branches located in more rural areas. These branches vary in terms of the services they offer, often operating with very limited operating hours—sometimes just a few hours per week—and offering a narrower range of medical services compared with full-scale primary care centres. Additionally, some regions may offer home healthcare services for individuals residing in areas with considerable distances to the nearest primary healthcare centre. However, we lack data on the extent of such branches and their specific locations. The omission of satellite branches and home healthcare services may lead to an underestimation of geographical accessibility in certain areas, particularly in rural or sparsely populated municipalities where satellite units often contribute to service provision. Future research should seek to incorporate information on satellite branches and outreach services to provide a more comprehensive assessment of primary care accessibility.

Another limitation is that travel times were estimated exclusively for car travel. Travel by car is not representative for all population groups, and individuals without access to a private vehicle or who are unable to drive may experience substantially different accessibility. Consequently, our results may overestimate effective accessibility for population groups with limited car access, including older adults, low-income individuals or residents in areas with weak transport infrastructure. Accordingly, our estimates should be interpreted as measures of potential geographical accessibility by private car rather than realised accessibility for all residents. Furthermore, the present study was descriptive and did not examine whether long travel times disproportionately affected specific population groups or socioeconomic areas. These important questions warrant further investigation in future studies.

## Conclusion

This study provides the first national, individual-level description of geographical accessibility to primary care centres in Sweden using driving distances and travel times. Overall, geographical accessibility was high: most residents lived close to their nearest primary care centre and could reach at least one provider within 10 min by car and very few faced travel times exceeding 30 min. At the same time, accessibility was unevenly distributed. Travel times and distances were strongly associated with the degree of municipal urbanisation, with markedly longer travel times in very rural and sparsely populated municipalities than in urban municipalities and large cities. Moreover, the scope for provider choice was substantially more limited outside urban areas, as distances to the second-nearest centre varied more than to the nearest provider, restricting opportunities to switch provider in remote settings.

Taken together, these findings suggest that Swedish primary care performs well on average in terms of geographic accessibility, but that important pockets of limited access remain—consistent with a geographical dimension of medical deserts. Policy efforts to strengthen equitable access should therefore prioritise areas with long travel times and limited provider choice, and complement geographical indicators with measures of staffing, waiting times and utilisation to better capture effective access. Future research should also incorporate satellite branches, home healthcare services and multimodal travel times to provide a more complete assessment of primary care accessibility across Sweden.

## Supplementary material

10.1136/bmjopen-2026-120220 online supplemental file 1

## Data Availability

Data may be obtained from a third party and are not publicly available.
