# Supplementary material for: Variations in geographical accessibility: a cross-sectional nationwide study of travel times in Swedish primary care
Source: BMJ Open. 2026 Sep 11;16(9):e120220. doi: 10.1136/bmjopen-2026-120220 (PMC13583706; doi:10.1136/bmjopen-2026-120220)
Supplement: Supplementary file 1 [file bmjopen-2026-120220.reviewer_comments.pdf]

PEER REVIEW HISTORY

BMJ Open publishes all reviews undertaken for accepted manuscripts. Reviewers are asked to complete a checklist review form and are provided with free text boxes to elaborate on their assessment. These free text comments are reproduced below.

ARTICLE DETAILS

Title (Provisional)

Variations in geographical accessibility: a cross-sectional nationwide study of travel times in Swedish primary care

Authors

Isaksson, David; Vengberg, Sofie; Fredriksson, Mio

VERSION 1 - REVIEW

**Reviewer**	**1**
**Name**	**Rotoli, Jason**
**Affiliation**	**University of Rochester Medical Center**
**Date**	**21-May-2026**
**COI**	**None**

Variation in geographical accessibility: A cross-sectional nationwide study of travel times in Swedish primary care

This study is a cross-sectional survey to examine variations in travel time to primary care centers by estimating driving time by car from different municipality type focusing on the supply-side (geographical accessibility) of care clinics. The study reports significant increases in time and travel distance beyond the closest primary care center. Additionally, there is a sign if ant amount of variation in time/distance in more rural areas. Most Swedish patients have access to their nearest primary care site in under 20 minutes; however, those in more rural areas may have lower likelihood of seeking care due to increased travel distance/time despite having high needs. Overall, this was one of the most well written papers I have had the opportunity to the review and I appreciate the authors' hard work.

ABSTRACT
- Concise and clear

INTRO
- Clear and concise, appropriately referenced, clear aim

METHODS
- Well written

- What is Statistics Sweden? Readers outside of Sweden may not be familiar and a brief summary and citation would be helpful

RESULTS
- Clear and concise
- Tables and figures are easy to read

DISCUSSION
- Clear, concise
- Nice brief comparison to other Nordic countries
- I appreciate the recognition that easy physical access to care (ie travel time and distance) does not always translate to healthcare utilization

---

Reviewer 2

Name Emamgholipour, Sara

Affiliation Tehran University of Medical Sciences School of Public Health, Department of Health Management and Economics

Date 13-Jun-2026

COI None

The subject of the paper (Geographical Access to Primary Care at the National Scale) is important and highly relevant. However, the following fundamental issues are evident in the manuscript:

1- In its current form, the manuscript resembles a descriptive geographical report rather than an analytical research article with a robust methodology.

2- It is unclear what added value the findings of the present study provide and which policy gaps or knowledge gaps they aim to address.

3- The definition and interpretation of geographical access in the article are incomplete. Geographical access has been equated solely with driving time to health centers, which is not an adequate or comprehensive measure.

4- in the introduction, the structure of primary care providers is described, noting that a proportion of centers are public and others private. However, the study does not examine whether there are differences in travel time and accessibility between these two sectors.

5- It should also be clarified how knowledge of driving time to these centers is intended to inform practice or policy. Is the objective to propose interventions to reduce travel time, or is the study merely reporting descriptive findings?

6- Despite the availability of relevant data, inequalities in access have not been examined to enable deeper analysis. Moreover, factors influencing travel time—such as age, gender, socioeconomic status, population density, and other relevant variables—have not been investigated.

7- Another issue overlooked in the study concerns control variables. While travel time by car is reported, the assumed average speed used in the calculations and the potential impact of speed variation on travel time are not specified. It is also unclear at what time of a day the travel time was calculated. Public transportation has not been considered, nor have weather conditions (e.g., snow and other adverse conditions).

8- It is not clear whether accessibility has been calculated for individuals without private vehicles or for older adults who do not drive.

9- Differences in access between deprived and advantaged areas have not been analyzed.

10-The disparity in access between older adults and the general population is an important issue, yet it has not been examined.

VERSION 1 - AUTHOR RESPONSE

Reviewer 1

The reviewer provided an overall very positive assessment of the manuscript and found the Abstract, Introduction, Results and Discussion to be clear and well written.

Response: We sincerely thank the reviewer for the very positive assessment of the manuscript and for the encouraging comments regarding the Abstract, Introduction, Results and Discussion. We greatly appreciate these remarks.

Comment: - What is Statistics Sweden? Readers outside of Sweden may not be familiar and a brief summary and citation would be helpful

Response: We thank the reviewer for this suggestion. We have added a brief description of Statistics Sweden in the Methods section, clarifying that it is Sweden's official government agency responsible for producing official national statistics.

Reviewer 2

The subject of the paper (Geographical Access to Primary Care at the National Scale) is important and highly relevant. However, the following fundamental issues are evident in the manuscript:

Comment 1- In its current form, the manuscript resembles a descriptive geographical report rather than an analytical research article with a robust methodology.

Response: We appreciate this comment and agree that the study is primarily descriptive. This was also the intention of the study. The objective was to provide the first nationwide description of geographical accessibility to Swedish primary care using individual-level travel time estimates. We have revised the Introduction and Discussion to clarify that the study is intended to establish a national baseline rather than identify determinants of accessibility.

Comment 2 - It is unclear what added value the findings of the present study provide and which policy gaps or knowledge gaps they aim to address.

Response: We thank the reviewer for this important comment. We agree that the contribution of the study should be stated more explicitly. We have therefore revised the Introduction and Discussion to better clarify the knowledge gap addressed by the study and its potential policy relevance. Although Sweden has universal primary care coverage, there has been no nationwide assessment of actual travel times to primary care centres. By quantifying geographical accessibility for the entire Swedish population, the present study provides an empirical baseline that can support healthcare planning, evaluation of structural reforms, and future studies of geographical inequalities in access.

Comment 3 - The definition and interpretation of geographical access in the article are incomplete. Geographical access has been equated solely with driving time to health centers, which is not an adequate or comprehensive measure.

Response: We agree that geographical accessibility is a broader concept than driving time alone and encompasses several dimensions. Our study examines one specific dimension of geographical accessibility—travel time by car—which is commonly used in health services research as an objective measure of travel-related accessibility. We have clarified throughout the manuscript that this represents only one dimension of geographical accessibility and have expanded the Limitations accordingly.

Comment 4 - in the introduction, the structure of primary care providers is described, noting that a proportion of centers are public and others private. However, the study does not examine whether there are differences in travel time and accessibility between these two sectors.

Response: We agree that comparisons between public and private providers could be of interest. However, this was outside the scope of the present study, whose objective was to describe geographical accessibility to all primary care centres irrespective of ownership. Information regarding ownership was included solely to describe the Swedish primary care system and not as an analytical variable in the present study.

Comment 5 - It should also be clarified how knowledge of driving time to these centers is intended to inform practice or policy. Is the objective to propose interventions to reduce travel time, or is the study merely reporting descriptive findings?

Response: We thank the reviewer for this helpful comment. We agree that the policy relevance of the findings should be stated more explicitly. We have therefore revised the Discussion to clarify how the results may inform healthcare planning. Specifically, we now state that the findings may support regional planning by identifying municipalities with comparatively long travel times and by providing a national benchmark for evaluating future changes in the geographical distribution of primary care services.

Comment 6 - Despite the availability of relevant data, inequalities in access have not been examined to enable deeper analysis. Moreover, factors influencing travel time—such as age, gender, socioeconomic status, population density, and other relevant variables—have not been investigated.

Response: We agree that analyses of socioeconomic inequalities and other determinants of accessibility are important. However, the purpose of the present study was descriptive rather than explanatory. Such analyses fall outside the scope of the current manuscript. To make this clearer, we have revised the Limitations section to explicitly state that the study was descriptive and did not

investigate inequalities between specific population groups or socioeconomic areas.

Comment 7 - Another issue overlooked in the study concerns control variables. While travel time by car is reported, the assumed average speed used in the calculations and the potential impact of speed variation on travel time are not specified. It is also unclear at what time of a day the travel time was calculated. Public transportation has not been considered, nor have weather conditions (e.g., snow and other adverse conditions).

Response: We thank the reviewer for this comment. We have clarified the Methods section by specifying that travel times were estimated using the default OSRM routing profile based on OpenStreetMap road network data and associated speed assumptions. We also clarify that the estimated travel times represent expected travel under typical driving conditions and therefore do not account for congestion, weather conditions, or temporary traffic disruptions.

Comment 8 - It is not clear whether accessibility has been calculated for individuals without private vehicles or for older adults who do not drive.

Response: We thank the reviewer for this important comment. Accessibility was estimated as driving travel time by private car for all residents and therefore represents potential geographical accessibility by private car, rather than realised accessibility for all population groups. We have clarified this limitation in the manuscript by explicitly stating that travel by car is not representative for all residents, that individuals without access to a private vehicle or who are unable to drive may experience substantially different accessibility, and that our estimates should therefore be interpreted as measures of potential geographical accessibility by private car rather than realised accessibility. We have also highlighted that future studies should investigate accessibility among specific population groups, including older adults and individuals with limited access to private transportation.

Comment 9 - Differences in access between deprived and advantaged areas have not been analyzed.

Response: We thank the reviewer for this comment and agree that socioeconomic inequalities in geographical accessibility are an important topic. However, the aim of the present study was to provide a descriptive nationwide assessment of geographical accessibility to primary care rather than to investigate determinants or inequalities in accessibility. We have clarified this in the Limitations section by explicitly stating that the study did not examine whether long travel times disproportionately affected specific population groups or socioeconomic areas, and that these important questions warrant further investigation in future studies.

Comment 10 - The disparity in access between older adults and the general population is an important issue, yet it has not been examined.

Response: We thank the reviewer for this comment. This concern is closely related to Comment 9. As noted above, the present study was designed as a descriptive nationwide assessment and did not investigate differences in accessibility between specific population groups. We have clarified this limitation in the manuscript and highlight accessibility among older adults as an important topic for future research.

We again thank the Editor and the Reviewers for their valuable comments and hope that the revisions satisfactorily address all concerns.
